# An audit of secondary peritonitis at a tertiary care university hospital of Sindh, Pakistan

**DOI:** 10.1186/1749-7922-7-6

**Published:** 2012-03-16

**Authors:** Ahmer A Memon, Faisal G Siddiqui, Arshad H Abro, Ahmed H Agha, Shahzadi Lubna, Abdul S Memon

**Affiliations:** 1 Department of Surgery, Liaquat University of Medical & Health Sciences, Jamshoro, 71000, Pakistan; 2 Department of Gynea & Obs, Liaquat University of Medical & Health Sciences, Jamshoro, 71000, Pakistan

**Keywords:** Ileostomy, Typhoid, Peritonitis

## Abstract

**Objective:**

Peritonitis is the most common life threatening surgical emergency, which requires urgent surgical intervention and is a significant cause of morbidity and mortality. The objective of this study was to highlight the frequency of secondary peritonitis and to analyze the site and causes of perforation, in our tertiary care setup.

**Methods:**

A retrospective analysis of 311 patients of secondary peritonitis was done from July 2008 to June 2010 at Liaquat University Hospital Jamshoro, Hyderabad, Sindh, Pakistan. All cases found to have peritonitis as a result of perforation of any part of gastrointestinal tract at the time of surgery were included in the study. All cases with either primary peritonitis or that due to anastomotic dehiscence were excluded.

**Results:**

A total of 311 patients were studied. Most of the patients were males (77%) and (89%) were in the third and fourth decades of life. Majority of the patients presented with pain (97%) associated with bowel symptoms. Most common site of perforation was small bowel (ileal 59%, jujenal 2%). In this series, most common risk factor of perforation was typhoid (43%). Ileostomy was the most commonly performed procedure. Overall morbidity was 48.5% and mortality was 17%.

**Conclusion:**

Considering the relatively higher rate of typhoid perforation quoted in this study, it is vital that typhoid fever ought to be eliminated by improved sanitation and immunizing programmes, otherwise surgeons will be confronted with its complications.

## Introduction

Generalized peritonitis is a common surgical emergency in developing countries [1]. Despite advances in surgical techniques, good antimicrobial therapy and intensive care support, it carries high morbidity and mortality while its management remains difficult and complex [2]. Peritonitis can be classified as primary, secondary or tertiary, depending upon the source of microbial contamination. Primary peritonitis is secondary to extra-peritoneal sources, the infection spreading mainly through haematogenous dissemination without visceral perforation. Secondary peritonitis, on the other hand, is caused by resident flora of the gastrointestinal or urogenital tracts, the organisms reaching peritoneum secondary to a mechanical break. Non-responding secondary peritonitis either due to failure of the host inflammatory response or overwhelming super infection leads to tertiary peritonitis [3].

Peritonitis, if not treated promptly, can lead to multisystem organ failure and death [4,5].

Current surgical treatment options include primary double-layered closure [6], segmental resection and anastomosis [7] and primary ileostomy [8,9].

This study aims to identify the causes, bacteriology and outcomes of different surgical methods for secondary peritonitis at Liaquat University Hospital.

## Material and methods

This retrospective study was conducted in Surgical Emergency Unit-I, Liaquat University Hospital, Hyderabad, Sindh, Pakistan over a period of two years from July 2008 to June 2010. Three hundred and eleven patients with acute abdomen, admitted through Accident and Emergency (A&E) Department were included in this study. The symptoms included abdominal pain, distension, vomiting and absolute constipation, dehydration and shock with an average of 3.5 days elapsing between onset of first symptom and admission to hospital. Based on history and physical examination, a provisional diagnosis of intestinal perforation was made which was confirmed by investigations including X-ray chest for pneumoperitoneum and abdominal X-ray for air fluid levels. All patients were resuscitated after passage of two 16-gauge cannulas, nasogastric tube and Foley's catheter. All patients received 2-3 l of Ringer's lactate and third generation cephalosporins (ceftriaxone) and quinolones (moxifloxacin), the later given in the last one year of study. With the confirmation of the initial diagnosis of intestinal perforation, emergency laparotomy was performed in all 311 patients. Perforations in the gastrointestinal tract were treated either with primary double-layered closure, segmental resection and anastomosis or loop ileostomy, depending upon the operative findings and general status of the patients. Peritoneal fluid was sent for culture and sensitivity in all patients. The peritoneal cavity was irrigated with an average of 2 l of warm normal saline and drains were left in abdomen and wound was closed either as mass closure or in layers depending upon the operator's choice. Patients were monitored post-operatively for recovery and early detection and management of complications.

Alvarado scoring was routinely done in our series in patients suspected to have peritonitis secondary to perforated appendicitis.

The study was given an approval by the institutional Ethical Review Committee (ERC).

## Results

Three hundred and eleven patients with diagnosis of acute abdomen were included in this study. There were 239 (77%) males and 72 (23%) females. The age ranged from 18 to 75 years with the maximum incidence (89%) in the third decade. Presenting symptoms included abdominal pain (97%), abdominal distension (91%), absolute constipation (80%) and vomiting (58%). All patients (100%) presented with dehydration and shock. Abdominal tenderness and rigidity were present 85 and 83% of the patients respectively. Various investigative findings are depicted in Table 1.

**Table 1:** Abnormalities on the initial investigations

Investigations	n = 311
Hyponatraemia(Na < 130 mEq/L)	173 (56%)
Hypokalemia(K < 2.7 mEq/L)	139 (45%)
Blood Urea Nitrogen(> 167 mg/dl)	104 (33%)
Serum Creatinine(< 1.7 mg/dl)	82 (26%)
Pneumoperitoneum on Chest X-Ray	164 (53%)
Air fluid levels on abdominal X-Ray	90 (29%)

All 311 patients underwent emergency laparotomy. In 182 (58%) cases, ileal perforation was the underlying cause for peritonitis. The second most common site of perforation was gastroduodenum, found in 56 (18%) patients. Other sites of perforation are shown in Table 2. The aetiology of perforations in 311 patients is depicted in Table 3.

**Table 2:** Site of perforation

Site of perforation	n = 311
Gastroduodenal	56 (18%)
- Duodenal	37 (11.9%)
- Gastic	19 (6.1%)
Jejunal	07 (2%)
Ileal	182 (59%)
Appendicular	47 (15%)
Colonic	19 (6%)

**Table 3:** Aetiology of perforation

Aetiology	(n = 311)
Typhoid	134 (43%)
Acid peptic disease	56 (18%)
Appendicular	47 (15%)
Tuberculosis	43 (13.8%)
Trauma	20 (6.4%)
Malignancy Ileocaecal Large bowel	11 (3.53%) 02 (0.64%) 09 (2.9%)

Two hundred and three (65%) cases were found to have generalized peritonitis while the remaining (35%) had localized peritonitis. Faecal exudate was seen in 243 (78%) patients while 68 (22%) cases had either clear or purulent exudate. The most commonly performed procedure in our series was ileostomy which was carried out in 81 (26%) patients, followed by simple closure in 73 (23%) patients. Other surgical procedures performed are depicted in Table 4. Postoperative complications were encountered in 143 (46%), cases (Table 5) especially in patients presenting late. The mean hospital stay ranged from 14 to 56 days. The morbidity and mortality in this series were 48.5 and 16.7%, respectively.

**Table 4:** Surgical procedure performed

Surgical procedure	(n = 311)
Ileostomy	81 (26%)
Simple closure	73 (24%)
Closure with Graham's patch (Omentopexy)	56 (18%)
Appendicectomy	47 (15%)
Resection and anastomosis	28 (9%)
Stricturoplasty	9 (3%)
Colostomy	17 (5%)

**Table 5:** Post operative complications

Complications	(n = 311)
Abdominal collection	13 (4.1%)
Wound infection	32 (10.2%)
Electrolyte imbalance	21 (6.7%)
Septicemia	33 (10.6%)
Burst abdomen	14 (4.5%)
Faecal fistula	19 (6.1%)
Ileostomy related complications	11 (3.5%)
Overall morbidity	151 (48.5%)
Mortality	52 (16.7%)

## Discussion

Generalized peritonitis is a frequently encountered emergency and remains a significant cause of morbidity and mortality which usually requires emergency surgery [10]. Worldwide there is a predominance of males presenting with this life-threatening disease [11,12]; our series also shows a similar trend, with a male to female ratio of 3.3:1.

Early diagnosis and treatment leads to improved results in terms of mortality. Majority of patients in our series presented late with the time interval between the onset of symptoms and admission varying from 12 hours to up to 6 days with an average of 3.5 days. Delay in seeking treatment associated with other factors such as malnourishment and impaired immunity was one of the major reasons for high mortality and morbidity in our series. Kaur N et al., in their study also attribute delay seeking surgical treatment as an important cause for high morbidity [13].

The diagnosis of the patients with peritonitis is clinical; all patients in our series presented with abdominal pain. The pain was sharp, insidious, constant and intense, and was aggravated with movements. Other symptoms included anorexia, nausea, vomiting, absolute constipation and abdominal distension. Langell JT and Mulvihill SJ report similar symptoms in their study [10].

Investigations in patients with peritonitis have dubious reliability. Only 164 (52.7%) patients in this series had evidence of pneumoperitoneum on x-ray chest. This corresponds well with another study, which reports pneumoperitoneum in 50% of cases with peritonitis [14]. Similarly, only 28.9% cases showed air fluid levels on x-ray abdomen.

In our study, distal gastrointestinal tract was the common site of perforation and was seen in 182 (58.5%) patients. This is corroborated by similar study by Quereshi AM [15] and Dorairajan LN [2] who report majority of perforations involving distal gastrointestinal tract such as ileum.

In our study, the most common cause of secondary peritonitis due to gastrointestinal tract perforation was typhoid which was found in 134(43%) cases; this was followed by peptic ulcer disease in 56(18%) cases. Duodenal perforation was more common (11.9%) compared to gastric perforation (6.1%). Chaterjee H too reported typhoid as the commonest cause of perforations in two separate studies [16,17].

We performed primary closure of the perforation in patients with typhoid peritonitis who were clinically stable and had minimal soling of the abdominal cavity. We selectively performed primary closure with proximal ileostomy in all other patients who presented late and had faecal contamination of peritoneal cavity, friable and gut and/or poor clinical condition, this is also supported by other studies [18-22].

Acid peptic disease was the second commonest cause of secondary peritonitis in our study being found in 56(18%) cases. These perforations were found either along the first part of the duodenum anteriorly (11.9%) or in the pylorus of the stomach (6.1%). These patients presented with the classical signs and symptoms of peritonitis, and required early surgery for a favourable outcome. We found that in such cases, closure of the perforation using a Graham's omental patch was a simple and safe procedure with low mortality, as supported by Subramanyam SG [23].

Dandpat MC studied 340 cases of Gastrointestinal perforations and found that 22(6.4%) patients developed secondary peritonitis secondary to perforated appendix [24]. However, in our series, secondary peritonitis due to appendicular perforations was the underlying cause in 47 (15%) of patients. Afridi SP had reported that the patients who developed secondary peritonitis due to perforated appendix present with the typical history of pain starting in the periumbilical region than shift to the right iliac fossa, or originated directly in the right iliac fossa and then spread to all over the abdomen [25]. We also observed that most of the patients with appendicular perforation presented in the similar manner. The patients with perforated appendix belonged to young age group.

Primary intestinal tuberculosis is uncommon in the west [26] but is still common in developing countries like Pakistan [27]. In our study, the clinical picture of the patients presenting with tuberculous perforation included symptoms such as abdominal pain, fever with night sweats and weight loss. Eighteen (5%) patients had history of subacute intestinal obstruction. Radiologic images revealed evidence of tuberculosis in 11(3.5%) patients. 19 (6%) of patients presented with peritonitis during the course of anti tuberculosis treatment. The commonest sites of involvement were terminal ileum and ileocaecal region though, multiple sites were also commonly found. Management of these patients included resection anastomosis of small gut, repair of perforation with ileoileal or ileo-transverse bypass, primary repair of perforation, stricturoplasty and right hemicolectomy. Biopsies were taken for histopathological examination from the edge of the perforation, omentum and mesenteric lymph nodes which proved the diagnosis of tuberculosis. Similar observations are reported by Akgun Y [28] and Serf R [29]. 11 cases of malignancy were found in our study. The majority of malignancies (9 cases) involved the large bowel, while 2 cases showed involvement of ileocaecal junction. All carcinomas were identified as adenocarcinomas on histopathology.

Surgical treatment of secondary peritonitis is highly demanding. Some authors have adopted laparoscopy as preferred surgical approach for the management of secondary peritonitis [30]. Laparoscopy is an emerging facility and in emergency setup, it is still in its infancy, being performed in only a few medical institutions of Pakistan. Due to the non-availability of laparoscopy in our emergency setup during the study period, no patient was treated laparoscopically.

In our study, postoperative complications included wound infection (28%), septicaemia (20%) and electrolyte imbalance (7%). However, postoperative complication in secondary peritonitis reported by Jhobta RS [10] are respiratory tract infections (28%), wound infection (25%), septicaemia (18%) and dyselectrolaemia (17%). Kim et al. [31] in their study report mortality rate of 9.9%. This is related to the delayed presentation of the patient to a definitive care hospital. In our study mortality rate was 16.7%. The high mortality in our setup could be attributed to the fact that this hospital caters to patients from far flung rural areas of the province. Illiteracy, low socio-economic status, improper infrastructure including inadequate transport and delayed referral to tertiary care hospital by the general practitioners are some of the reasons for these patients coming late to our medical facility.

## Conclusion

The presentation of secondary peritonitis in Pakistan continues to be different from its western counterpart. The In majority of cases the presentation to the hospital was late with well established generalized peritonitis with purulent/fecal contamination and varying degree of septicemia. Good pre-operation assessment and early management will decrease the morbidity, mortality and complications of secondary peritonitis.

## Competing interests

The authors declare that they have no competing interests.

## Authors' contributions

AAM carried out acquisition, analysis, interpretation of the data and drafting of the manuscript. FGS was involved interpretation of the data, drafting of the manuscript, and revised it critically for the intellectual content till the final version was reached. AHA, AHA, SL and ASM have read, edited and approved the final manuscript. All authors read and approved the final manuscript.

## References

[B1] Adesunkanmi ARK Badmus TA Fadiora FO Agbakwuru EA Generalized peritonitis secondary to typhoid ileal perforation: Assessment of severity using modified APACHE II score Indian J Surg 2005 67 29 33

[B2] Dorairajan LN Gupta S Deo SV Chumber S Sharma L Peritonitis in India-a decade's experience Trop Gastroenterol 1995 16 1 33 38 7645051

[B3] Ordonez CA Puyana JC Management of peritonitis in the critically ill patient Surg Clin North Am 2006 86 6 1323 1349 10.1016/j.suc.2006.09.006 17116451 PMC3413265

[B4] Gupta S Kaushik R Peritonitis--the Eastern experience World J Emerg Surg 2006 1 13 10.1186/1749-7922-1-13 16759427 PMC1475566

[B5] Eid HO Hefny AF Joshi S Abu-Zidan FM Non-traumatic perforation of the small bowel Afr Health Sci 2008 8 1 36 39 19357730 PMC2408541

[B6] Athie CG Guizar CB Alcantara AV Alcaraz GH Montalvo EJ Twenty-five years of experience in the surgical treatment of perforation of the ileum caused by Salmonella typhi at the General Hospital of Mexico City, Mexico Surgery 1998 123 6 632 636 10.1016/S0039-6060(98)70201-6 9626313

[B7] Kaul BK Operative management of typhoid perforation in children Int Surg 1975 60 8 407 410 1158618

[B8] Singh KP Singh K Kohli JS Choice of surgical procedure in typhoid perforation: experience in 42 cases J Indian Med Assoc 1991 89 9 255 256 1795109

[B9] Khalid S Irfan A Outcome of ileostomy in cases of typhoid perforation presenting after 48 hours J Rawal Med Coll 2000 4 17 19

[B10] Langell JT Mulvihill SJ Gastrointestinal perforation and the acute abdomen Med Clin North Am 2008 92 3 599 625 10.1016/j.mcna.2007.12.004 18387378

[B11] Jhobta RS Attri AK Kaushik R Sharma R Jhobta A Spectrum of perforation peritonitis in India-review of 504 consecutive cases World J Emerg Surg 2006 1 26 10.1186/1749-7922-1-26 16953884 PMC1570451

[B12] Wani RA Parray FQ Bhat NA Wani MA Bhat TH Farzana F Nontraumatic terminal ileal perforation World J Emerg Surg 2006 1 7 10.1186/1749-7922-1-7 16759405 PMC1459268

[B13] Kaur N Gupta MK Minocha VR Early enteral feeding by nasoenteric tubes in patients with perforation peritonitis World J Surg 2005 29 8 1023 1027 discussion 7-8 10.1007/s00268-005-7491-z 15981045

[B14] Conroy JV Acute ileitis with ulceration and perforation due to paratyphoid fever; report of eighty-five cases Mil Med 1957 120 2 79 92 13399825

[B15] Qureshi AM Zafar A Saeed K Quddus A Predictive power of Mannheim Peritonitis Index J Coll Physicians Surg Pak 2005 15 11 693 696 16300704

[B16] Chatterjee H Jagdish S Pai D Satish N Jayadev D Reddy PS Changing trends in outcome of typhoid ileal perforations over three decades in Pondicherry Trop Gastroenterol 2001 22 3 155 158 11681112

[B17] Chatterjee H Pai D Jagdish S Satish N Jayadev D Srikanthreddy P Pattern of nontyphoid ileal perforation over three decades in Pondicherry Trop Gastroenterol 2003 24 3 144 147 14978991

[B18] Adesunkanmi AR Ajao OG The prognostic factors in typhoid ileal perforation: a prospective study of 50 patients J R Coll Surg Edinb 1997 42 6 395 399 9448395

[B19] Maurya SD Gupta HC Tiwari A Sharma BD Typhoid bowel perforation: a review of 264 cases Int Surg 1984 69 2 155 158 6500881

[B20] Meier DE Imediegwu OO Tarpley JL Perforated typhoid enteritis: operative experience with 108 cases Am J Surg 1989 157 4 423 427 10.1016/0002-9610(89)90591-6 2929866

[B21] Archampong EQ Tropical diseases of the small bowel World J Surg 1985 9 6 887 896 10.1007/BF01655393 4082610

[B22] Eustache JM Kreis DJ Jr Typhoid perforation of the intestine Arch Surg 1983 118 11 1269 1271 10.1001/archsurg.1983.01390110027007 6639337

[B23] Subramanyam SG Sunder N Saleem KM Kilpadi AB Peritonitis in patients over the age of 50 years: 98 cases managed surgically Trop Doct 2005 35 4 247 250 10.1258/004947505774938611 16354492

[B24] Dandapat MC Mukherjee LM Mishra SB Howlader PC Gastrointestinal perforations Indian J Surg 1991 53 189 193

[B25] Afridi SP Malik F Ur-Rahman S Shamim S Samo KA Spectrum of perforation peritonitis in Pakistan: 300 cases Eastern experience World J Emerg Surg 2008 3 31 10.1186/1749-7922-3-31 18992164 PMC2614978

[B26] Michalopoulos A Papadopoulos VN Panidis S Papavramidis TS Chiotis A Basdanis G Cecal obstruction due to primary intestinal tuberculosis: a case series J Med Case Reports 2011 5 128 10.1186/1752-1947-5-128 21450062 PMC3073921

[B27] Jamal S Khan Z Ahmed I Shabbir S Khaliq T Presentation and Outcome of Abdominal Tuberculosis in a Tertiary Care Unit Ann Pak Inst Med Sci 2011 7 1 33 36

[B28] Akgun Y Intestinal and peritoneal tuberculosis: changing trends over 10 years and a review of 80 patients Can J Surg 2005 48 2 131 136 15887793 PMC3211607

[B29] Sefr R Rotterova P Konecny J Perforation peritonitis in primary intestinal tuberculosis Dig Surg 2001 18 6 475 479 10.1159/000050197 11799299

[B30] Ramachandran CS Agarwal S Dip DG Arora V Laparoscopic surgical management of perforative peritonitis in enteric fever: a preliminary study Surg Laparosc Endosc Percutan Tech 2004 14 3 122 124 10.1097/01.sle.0000129387.76641.29 15471016

[B31] Kim JP Oh SK Jarrett F Management of ileal perforation due to typhoid fever Ann Surg 1975 181 1 88 91 10.1097/00000658-197501000-00019 1119873 PMC1343721

